# The Coming-of-Age of Subcutaneous Injectable Contraception

**DOI:** 10.9745/GHSP-D-18-00050

**Published:** 2018-03-21

**Authors:** Kimberly Cole, Abdulmumin Saad

**Affiliations:** a United States Agency for International Development, Global Health Support Initiative-III, Washington, DC, USA.; b United States Agency for International Development, Global Health Fellows Program II, Washington, DC, USA.

## Abstract

DMPA-SC is a contraceptive injectable formulation that provides women and couples another important voluntary family planning option. It offers characteristics that many women like, including cost and time savings, and has the potential to be delivered by a range of health care cadres in a variety of service delivery channels.

See related articles by Stout et al. and by Georges et al.

Thirty-one years ago, Uniject—a prefilled, single-dose syringe and needle package that features a collapsible blister—was conceptualized.^1^ Seventeen years later Uniject was approved to administer 104 mg of the contraceptive depot medroxyprogesterone acetate subcutaneously (DMPA-SC).^2^ DMPA-SC is still nascent in many countries, but in others it has transitioned to prominence even where there is already an intramuscular DMPA (DMPA-IM) product on the market. DMPA-SC is now coming of age, and offering it alongside a broad range of other contraceptive options, including fertility awareness methods, long-acting reversible methods, and permanent methods, increases choice and access to voluntary family planning.

This issue of GHSP includes 2 articles that present data on DMPA-SC introduction experiences in 4 countries that were among the earliest to introduce DMPA-SC and have shown great progress: Burkina Faso (Stout et al.^3^ and Georges et al.^4^), and Niger, Senegal, and Uganda (Stout et al.^3^).

## THE CONTEXT

In developing regions, 214 million women of reproductive age want to avoid pregnancy but are not using a modern contraceptive method.^5^ The Family Planning 2020 (FP2020) global partnership has set an ambitious goal to reach more than half of these women with voluntary family planning, yet we are not on track to achieve this goal.^6^^,^^7^ Given the great challenge, adding another voluntary contraceptive option to the method mix will help women and couples to optimally time and space their pregnancies for the safest and healthiest outcomes. It cannot be overemphasized that voluntarism, informed choice, and a respect for clients' rights must be central to any family planning program.

## WHAT'S NEW?

Globally, there is a strong association between the range of voluntary contraceptive choices and contraceptive use: use increases when more methods are available and also when current methods are improved.^8^ DMPA-SC is an improvement upon the intramuscular DMPA formulation. The subcutaneous formulation features a 30% lower dose of DMPA, yet provides the same efficacy and length of protection as DMPA-IM.

In a growing number of countries, the client herself can self-inject. Where women can self-inject, DMPA-SC offers the most effective woman-controlled contraceptive option available.

It is important that providers counsel clients on all methods they might wish to discuss. It is also important that both provider and client understand the differences between DMPA-SC and DMPA-IM (Table).

**TABLE.** A Comparison Between DMPA-SC and DMPA-IM Injectables

Characteristic	DMPA-SC	DMPA-IM
Formulation	104 mg/0.65 mL of DMPA in the Uniject injection system; all-in-one Uniject system simplifies procurement and logistics	150 mg/mL of DMPA, administered by intramuscular injection, available in vials or prefilled syringes
Needle	3/8” needle; 23 gauge ultra-thin	1” needle; 22 gauge with a 21–23 gauge range option
Administration	Where permitted, can be administered by CHWs, pharmacists, or by the woman herself	Typically administered by a provider, but can be administered by CHWs and pharmacists where permitted
Shelf life	3 years	5 years
Efficacy	99% contraceptive efficacy	
Safety	Similar safety profile	
Duration and mechanism of action	Provides 3 months of contraceptive protection per dose by preventing ovulation and thickening cervical mucus	
Safety during breastfeeding	Safe for breastfeeding mothers at 6 weeks postpartum	
Health benefits	Reduces the risk of endometrial and ovarian cancer Protects from uterine fibroids, endometrial cancer, ectopic pregnancy, and symptomatic pelvic inflammatory disease May reduce sickle cell crises in some women with sickle cell anemia Prevents seizures in some women with epilepsy Prevents iron deficiency anemia in some women	
Side effects	May cause headaches, bleeding irregularities, weight gain, injection-site reactions	
Protection against HIV or other STIs	Does not protect against HIV or other STIs	

Abbreviations: CHW, community health worker; DMPA, depot medroxyprogesterone acetate; DMPA-IM, intramuscular DMPA; DMPA-SC, subcutaneous DMPA; STI, sexually transmitted infection.

Source: Spieler (2010)^1^ and Family Health International (2010).^9^

## DMPA-SC HAS THE POTENTIAL TO REACH MORE CLIENTS AND IMPROVE SATISFACTION

Both articles included in this issue of GHSP demonstrate that DMPA-SC offers more women (especially those who face barriers when interacting with the health system) access to a new voluntary contraceptive method that could meet their needs and reproductive intentions. Of the 120 million women that FP2020 seeks to reach, 75 million have never used a contraceptive method (never-users) and 45 million have used a method in the past but have discontinued (discontinuers).^10^^,^^11^

DMPA-SC has been shown through introduction experiences, such as the ones described in this issue,^3^ to be attractive to never-users. Like past studies, the Stout article was able to show that many new acceptors of voluntary family planning (i.e., never-users) have shown a preference for DMPA-SC. New acceptors often include younger clients, and younger clients may prefer DMPA-SC if it is available closer to their homes and because the needle is smaller than the intramuscular needle, although proximity and needle size are traits that many users find attractive.

Previous studies have also established the acceptability of DMPA-SC, and many clients prefer it to other methods.^12^^,^^13^ One reason that clients are attracted to DMPA-SC is the cost and time savings that it offers. In community-based distribution settings, a woman wouldn't need to travel to a clinic since it is offered in her community. In self-injection settings, clients are often given 2 to 3 doses, reducing the number of trips they would need for resupply.

DMPA-SC may also ameliorate the high contraceptive discontinuation rates that are typical of injectables. The typical discontinuation rate at 12 months for DMPA-IM is 40% to 50%, but studies have found that DMPA-SC self-injectors have a more than 50% increase in continuation through 12 months compared with a provider-administered injection.^14^^–^^16^

Program data demonstrate that the process of introducing DMPA-SC into the method mix can increase voluntary uptake of contraceptive methods overall, not just of DMPA-SC.^17^ This is likely happening because when programs are introducing DMPA-SC they are taking the opportunity to retrain providers on all voluntary family planning methods and reinforcing the importance of voluntarism and informed choice.

Introducing DMPA-SC into the method mix can increase voluntary uptake of contraceptive methods overall, not just of DMPA-SC.

## ADVANCING ACCESS AND QUALITY

DMPA-SC can be programmed in a health system through a variety of delivery channels. By introducing the product at different levels and types of health facilities, in pharmacies and drug shops, and through community health workers, clients have more voluntary contraceptive options. In most parts of the world, community-based family planning programs and the private sector are important segments of the market.^18^^,^^19^ DMPA-SC is an ideal product for these sectors, but it requires an enabling environment for success. The articles in GHSP highlight the importance of task sharing. This product has been shown to be especially acceptable and in demand at the community level and through pharmacies. Task sharing can increase contraceptive access by expanding the range of methods that community health workers, lay health workers, and pharmacists can offer.

Programs have faced common challenges that include ensuring high-quality training and adequate supportive supervision. Misunderstandings and inconsistencies, even among experienced providers, may persist even after training.^20^ Additional coaching at both the facility and community levels can mitigate this weakness. Providers often need additional time and support to become comfortable counseling on new methods.

## PROGRAMMING TAKEAWAYS FOR SUCCESSFUL INTRODUCTION AND SCALE UP OF DMPA-SC

The Stout article describes a variety of different introduction approaches, illustrating the many options a country may consider. Globally, countries tend to co-position DMPA-SC alongside DMPA-IM, transition from IM to SC, or roll out targeted introduction by piloting different approaches. There is no “right” introduction approach; country-level decisions around programming and procurement of contraceptive methods are complex, involve multiple stakeholders, and require thoughtful planning. However the intended outcome should be that more women have voluntary access to this method if it meets their needs.

The Box summarizes some of the conditions necessary for successful introduction, many drawn from the Stout and Georges articles.

BOX **Elements Promoting Successful Introduction of DMPA-SC**

**Policy**

- Encourage strong Ministry of Health leadership.
- Promote task sharing: Countries can achieve high impact without including task sharing, but policies that allow for community health worker or pharmacist administration and/or self-injection maximize its potential.

**Service Delivery**

- Use a rapid, cascade approach to provider training.
- Counsel on all voluntary family planning methods, including those available through referral while ensuring comprehensible information is provided on the method chosen.
- Counsel on the method's characteristics including bleeding changes as well as the need for simultaneous use of condoms for dual protection to prevent HIV and other sexually transmitted infections.
- Offer the method through community channels, mobile outreach, and the private sector, supported by extensive demand-generation activities.

**Integration**

- Integrate with maternal and child health and other health and non-health services.
- Quickly make DMPA-SC a normal part of commodity planning to increase commodity security and leverage existing distribution systems.

**Monitoring and Evaluation**

- Disaggregate health information system data by injectable type (IM vs. SC) and collect data more frequently than semiannually.
- Disaggregate users by age to better understand user dynamics, and by prior contraceptive use to track new users.
- Share data openly, especially between the public and private sectors.

## PROGRAMMING UNKNOWNS AND WORDS OF CAUTION AROUND HIV

There is evidence of a possible increased risk of acquiring HIV among progestin-only injectable users. Uncertainty exists about whether this is due to methodological issues with the evidence or to a real biological effect.^21^ Currently there are no epidemiological data available on possible association between DMPA-SC specifically and risk of acquiring HIV. On March 2, 2017, the World Health Organization, in its *Medical Eligibility Criteria for Contraceptive Use*, changed use of DMPA injectable products among women at high risk of HIV acquisition from category 1 to category 2.^22^ This means that for women at high risk of HIV, the advantages of using DMPA products generally outweigh the theoretical or proven risk. Women should not be denied progestin-only injectables because of concerns about the possible increased risk of HIV. Rather, women considering progestin-only injectables should be advised about these concerns, about the uncertainty over whether there is a causal relationship, and about how to minimize their risk of acquiring HIV, including correct and consistent use of condoms, antiretroviral therapy initiation for partners living with HIV where appropriate, and pre-exposure prophylaxis where available. A wide range of voluntary family planning methods must be available, and when introducing a new method such as DMPA-SC, consideration should be given to retraining providers on clinical and counseling skills for all contraceptive methods and HIV risks.^23^^,^^24^

Given the inconclusive data, the question of whether DMPA increases women's risk of HIV is a critical public health issue requiring the strongest evidence possible. The ongoing Evidence for Contraceptive Options and HIV Outcomes (ECHO) study is designed to fill this gap and provide robust evidence on the relative risks (HIV acquisition) and benefits (pregnancy prevention) between 3 effective contraceptive methods (DMPA-IM; levonorgestrel implant; copper intrauterine device).^25^ It is important to note that the study does not include DMPA-SC, but the results may affect the introduction and rollout of DMPA-SC.

## ACCESSIBILITY OVER THE LONG TERM

Countries and implementers understandably want long-term access to affordable DMPA-SC before initiating a program at scale. For the DMPA-SC product marketed under the brand name Sayana Press and manufactured by Pfizer, the current price is $0.85 per dose for the next 6 years in the 69 FP2020 countries. Those countries can currently procure DMPA-IM for $0.88 per dose, or less. A partnership of global donors and other stakeholders is committed to ensuring long-term sustainability and access to an affordable DMPA-SC product. These organizations are working toward ensuring a healthy market for DMPA-SC supply, including supplier diversity, sufficient demand, and increasingly affordable pricing for DMPA-SC in FP2020 countries.

Another requirement for long-term accessibility is supply chain security. Supply chain systems should be strengthened to mitigate negative outcomes (stock-outs occurred in half of the country experiences described in this issue). The product itself enables simplified logistics because of its all-in-one packaging. This translates into easier transportation and storage due to reduced weight and volume, and there is less waste to dispose. To strengthen commodity security, the Stout article offers the Senegal experience where stock-outs were negligible due in part to the Informed Push Model.

DMPA-SC enables simplified logistics because of its all-in-one packaging.

## CONCLUSION

Decades of research and development led to the approval of DMPA-SC approximately 14 years ago. This product is now coming of age. Countries are adding it to their basket of voluntary contraceptive methods so more women will have access to a new choice. As more women of reproductive age learn about healthy timing and spacing of pregnancies, they are well served by the affordable availability of better and more contraceptive options to enable them to achieve their desired family size. DMPA-SC is one more option to help them do it.

## Notes

**Funding:** None.
